# Rapid Screening of *Aedes aegypti* Mosquitoes for Susceptibility to Insecticides as Part of Zika Emergency Response, Puerto Rico

**DOI:** 10.3201/eid2510.181847

**Published:** 2019-10

**Authors:** Ryan R. Hemme, Lucrecia Vizcaino, Angela F. Harris, Gilberto Felix, Michael Kavanaugh, Joan L. Kenney, Nicole M. Nazario, Marvin S. Godsey, Roberto Barrera, Julieanne Miranda, Audrey Lenhart

**Affiliations:** Centers for Disease Control and Prevention, San Juan, Puerto Rico, USA (R.R. Hemme, A.F. Harris, G. Felix, R. Barrera);; Centers for Disease Control and Prevention, Atlanta, Georgia, USA (L. Vizcaino, A. Lenhart);; United States Navy, Jacksonville, Florida, USA (M. Kavanaugh);; Centers for Disease Control and Prevention, Fort Collins, Colorado, USA (J.L. Kenney, M.S. Godsey);; Puerto Rico Vector Control Unit, San Juan (N.M. Nazario, J. Miranda)

**Keywords:** Zika virus, Aedes aegypti, insecticide resistance, Puerto Rico, mosquitoes, viruses, vector-borne infections, arboviruses, Puerto Rico, United States

## Abstract

In response to the 2016 Zika outbreak, *Aedes aegypti* mosquitoes from 38 locations across Puerto Rico were screened using Centers for Disease Control and Prevention bottle bioassays for sensitivity to insecticides used for mosquito control. All populations were resistant to pyrethroids. Naled, an organophosphate, was the most effective insecticide, killing all mosquitoes tested.

The first case of autochthonous Zika virus infection in the Western Hemisphere was reported in Brazil in 2015, followed by reports from 26 countries and territories in the Caribbean and Central and South America (*1*,*2*). On December 31, 2015, the Puerto Rico Department of Health reported the first autochthonous case of Zika virus infection, and by the end of 2016, ≈35,000 cases had been reported (*1*,*3*).

During 1945–1955, as part of a broader campaign to eliminate *Aedes aegypti* mosquitoes from the Western Hemisphere, vector-control efforts used DDT to control these mosquitoes in Puerto Rico, primarily through residual treatments in houses; resistance in *Ae. aegypti* mosquitoes to organochlorine insecticides (DDT and dieldrin) was reported as early as 1961 (*4*,*5*). The first reports of resistance to organophosphate insecticides were published in the 1970s, and by the 1980s, *Ae. aegypti* mosquitoes in Puerto Rico had developed resistance to synthetic pyrethroids (*5*,*6*). At the time of the Zika virus outbreak, vector-control authorities in Puerto Rico used cleanup campaigns, community education, and adulticiding with ultralow-volume (ULV) truck-mounted sprayers to control *Ae. aegypti* mosquitoes. Commercial products containing the pyrethroid insecticide permethrin were most commonly used in ULV applications, and a few municipalities used one that contained a proprietary mixture of botanical compounds. The heavy reliance on permethrin-based products raised concerns, given recent reports that *Ae. aegypti* populations in 8 municipalities were already highly resistant to it (*7*).

During outbreaks, vector-control strategies must rapidly suppress adult mosquito populations to interrupt disease transmission (*8*,*9*). In March 2016, in response to the Zika virus outbreak, the World Health Organization released special guidance on reducing human–vector contact, recommending the use of targeted residual spraying and ULV spraying against adult mosquitoes; larval control, including source reduction; and personal protective measures, including the use of topical repellents (*8*,*9*). To learn which adult mosquito-control products would have the greatest likelihood of rapidly suppressing *Ae. aegypti* mosquito populations, during early 2016 we conducted an emergency islandwide screening in Puerto Rico for susceptibility to insecticides in products available for public health use in areas of active or at high risk for Zika virus transmission.

## The Study

Sampling sites for this investigation comprised municipalities with large urban populations and other potential areas at high risk for Zika virus transmission islandwide. We collected *Ae. aegypti* eggs from 38 neighborhoods (clusters of ≈200 houses) within 23 municipalities in Puerto Rico (Figure) using standard black ovitraps containing 10% hay infusion and seed germination paper as the oviposition substrate (*10*). We placed 2–4 ovitraps at homes within sampling neighborhoods (120 ovitraps per neighborhood) after acquiring verbal consent from the homeowners. Ovitraps remained in the field for 4 days, after which they were retrieved and the germination papers containing eggs were dried until hatching.

**Figure:**
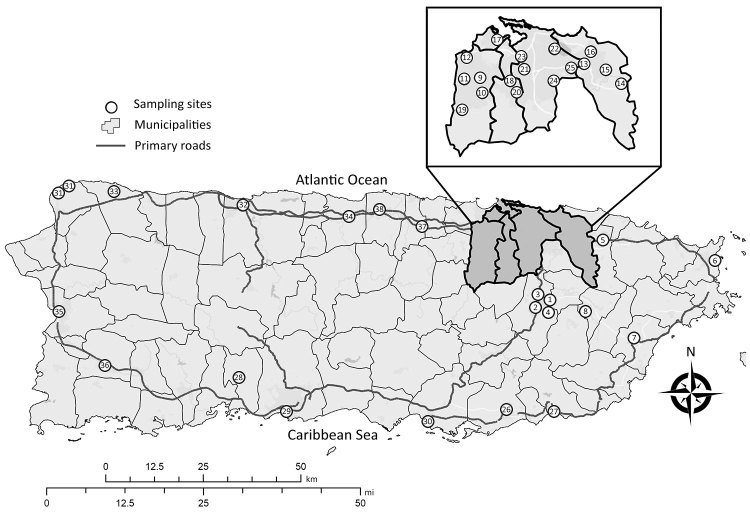
Locations of *Aedes aegypti* egg collections for insecticide resistance testing, Puerto Rico, 2016. Municipalities or barrios: 1, Caguas, Condado Moderno; 2, Caguas, Urb Idamaris Garden; 3, Caguas, Villa Blanca; 4, Caguas, Villa de Castro; 5, Canóvanas; 6, Fajardo; 7, Humacao; 8, Juncos; 9, Bayamón; 10, Bayamón, Irlanda; 11, Bayamón, Pájaros; 12, Bayamón, Teresita; 13, Carolina, El Comandante; 14, Carolina, Los Colobos; 15, Carolina, Villa Carolina; 16, Carolina, Vistamar; 17, Cataño; 18, Guaynabo, Ponce de León; 19, Guaynabo, Sabana; 20. Guaynabo, Villa Clementina; 21, San Juan, Caparra Terrace; 22, San Juan, Israel; 23, San Juan, Puerto Nuevo; 24, San Juan, Venus Garden; 25, San Juan, El Commandante; 26, Guayama; 27, Patillas; 28, Peñuelas; 29, Ponce; 30, Salinas; 31, Aquadilla; 32, Arecibo; 33, Isabela; 34, Manati; 35, Mayagüez; 36, San German; 37, Vega Alta; 38, Vega Baja. Inset shows closer view of dark gray shaded area.

We hatched eggs and reared mosquitoes under insectary conditions using standardized protocols (*11*). When the mosquitoes were 2–5 days old, we evaluated non–blood-fed females for resistance to insecticides using the standard Centers for Disease Control and Prevention bottle bioassay protocol (*12*). We screened populations for susceptibility to 11 insecticides and scored mortality at a 30-minute diagnostic time for all insecticides (Table). We used the insecticide-susceptible Rockefeller strain of *Ae. aegypti* mosquitoes as a control. We conducted bioassays during March–June 2016; partial findings were initially published on the Centers for Disease Control and Prevention website in April 2016 (https://www.cdc.gov/zika/vector/testing-puertorico.html).

**Table:** Locations and death rates for *Aedes aegypti* mosquitoes in bottle bioassays, Puerto Rico, 2016

Municipality or barrio	Insecticide, concentration per bottle												
	Simple pyrethroids, 15 μg						Cyano-pyrethroids, 10 μg				Organophosphates		Carbamate, bendiocarb, 12.5 μg
	Permethirn	Phenothrin	Etofenprox	Tetramethrin	Bifenthrin		Alpha-cypermethrin	Deltamethrin	Lambda-cyhalothrin		Naled, 25 μg	Malathion, 50 μg	
East													
Caguas, Condado Moderno					3.7		27.3	25.0	5.9		100	66.7	
Caguas, Urb. Idamaris Garden	5.0							90.4					
Caguas, Villa Blanca	0							87.2					
Caguas, Villa de Castro	0							75.9					
Canóvanas		1.2		0	20.0		44.1	90.0	16.0				
Fajardo	0	0		0	27.5		38.1	60.0	41.5		**100**	53.7	
Humacao	0	0		0	22.5		23.5	62.5	36.8		**100**	67.3	
Juncos	0				45.1		44.3	73.1	65.9		**100**	58.8	
Metro													
Bayamón	0						19.2	86.7	33.3		**100**		
Bayamón, Irlanda								31.6					
Bayamón, Pájaros								50.0					
Bayamón, Teresita								69.6					
Carolina, El Comandante								1.1					
Carolina, Los Colobos								0					
Carolina, Villa Carolina								1.0					
Carolina, Vistamar								22.5					**100**
Cataño	0						60.8	82.7	64.0		**100**		
Guaynabo, Ponce de León					0		5.9	66.7	40.0		**100**	12.8	
Guaynabo, Sabana								78.2					**100**
Guaynabo, Villa Clementina								55.0					**100**
San Juan, Caparra Terrace								0					**100**
San Juan, Israel								0					**100**
San Juan, Puerto Nuevo	0				29.4		59.0	92.8	47.2		**100**	53.1	
San Juan, Venus Garden								2.1					**100**
San Juan, El Commandante		0		0	14.3		39.6	97.5	45.9				
South													
Guayama		1.4	1.3		90.0		**98.7**	**100**	93.7				
Patillas	0				82.9		68.6	46.0	78.0		**100**	80.8	
Peñuelas		0			47.3		48.5	79.4	60.8		**100**	40.5	
Ponce		2.1			2.2		40.0	27.9	34.1		**100**	34.0	
Salinas		0	0		82.5		95.7	**100**	72.5				
West													
Aquadilla					0		31.8	52.6	18.2		**100**		
Arecibo					7.7		85.5	**98.8**	40.3				
Isabela					45.1		82.4	**100**	86.6		**100**	25.0	
Manati					3.8		84.0	96.6	93.8		**100**	93.8	
Mayagüez					7.8		84.0	96.0	89.0				
San German							15.6	83.8	20.0				
Vega Alta	0						46.9	93.6	48.6				
Vega Baja	0						43.0	**98.0**					

*Bold indicates insecticides that are effective against *Ae. aegypti* mosquitoes as defined by the World Health Organization (susceptible as >98% mortality, partially resistant as 90%–97% mortality, and resistant as <90% mortality) (*12*). Blank cells indicate insecticide resistance assays for thos insecticides with those mosquitoes (from the corresponding geographic areas) were not done.

The World Health Organization recommends that insecticide resistance, partial resistance, and susceptibility for mosquito populations are interpreted from bioassay data at <90%, 91%–98%, and >98% mortality, respectively. All populations we tested were resistant to permethrin, and results of initial testing with phenothrin, etofenprox, and tetramethrin suggested that populations were highly resistant across the simple pyrethroids (Table). Because these preliminary findings suggested that simple pyrethroids would not be effective against *Ae. aegypti* mosquitoes in Puerto Rico, we did not test all populations against this class of insecticide, opting instead to focus on screening alternatives. Of the 3 cyano-pyrethroids tested, deltamethrin was effective in more populations; we found fully susceptible *Ae. aegypti* populations in 5 municipalities, and results from 4 additional municipalities showed partial resistance (Table). Overall, the organophosphate naled was the most promising insecticide tested; all *Ae. aegypti* populations showed 100% susceptibility (Table). However, efforts to launch a naled-based response to the Zika epidemic led to strong public opposition and were ultimately canceled. Although currently no product containing bendiocarb is registered for public health use by the US Environmental Protection Agency, we screened its effectiveness against *Ae. aegypti* mosquitoes as an alternative insecticide in 6 populations, and all were susceptible (Table).

## Conclusions

The primary objective of our survey was to rapidly screen key *Ae. aegypti* mosquito populations in Puerto Rico for susceptibility to insecticides that could be quickly deployed to address the Zika outbreak. Our results strongly suggested that the use of simple pyrethroids should be avoided because of widespread insecticide resistance. Results from the cyano-pyrethroid and malathion assays were less straightforward because resistance was geographically heterogeneous. This survey did not include mosquito populations from all municipalities; therefore, the resistance profiles of *Ae. aegypti* mosquitoes from large portions of Puerto Rico remain unknown, making islandwide policy recommendations difficult. Furthermore, the high degree of fine-scale spatial heterogeneity in the resistance profiles indicated that a mosaic insecticide treatment strategy that applied different products in different locations based on their resistance profile would be logistically challenging.

This study illustrates the challenges in translating laboratory findings into actionable vector-control strategies in the field, especially during an arbovirus outbreak. We did not find, as hoped, 1 insecticide effective at killing *Ae. aegypti* adults islandwide in Puerto Rico and available to municipalities for ground-based ULV spraying. The most effective insecticide, naled, can be applied only from the air, according to its Environmental Protection Agency label. In addition to concerns about insecticide efficacy and acceptability, products can vary greatly in their cost. For example, switching from a permethrin-based product to a deltamethrin-based product for use in truck-mounted ULV spraying would substantially increase program costs. The most commonly used permethrin product that is commercially available in Puerto Rico carries a local cost of $0.55 per acre, whereas a commercial product containing deltamethrin costs $1.99 per acre to apply, a cost increase of 260%.

Our results provide a rapid snapshot of resistance to key insecticides across Puerto Rico during the Zika emergency response. The findings highlight the importance of collecting routine data on insecticide resistance to develop vector-control strategies based on evidence from long-term trends. Routine and systematic surveillance of insecticide resistance should be used to guide vector-control policies for outbreak response and routine vector control. These results also underscore the importance of vector-control approaches that do not rely on insecticides as part of an integrated vector management strategy for Puerto Rico.
